# The Clinical Correlations of *Helicobacter pylori* Virulence Factors and Chronic Spontaneous Urticaria

**DOI:** 10.1155/2013/436727

**Published:** 2013-07-16

**Authors:** Yi-Chun Chiu, Wei-Chen Tai, Seng-Kee Chuah, Ping-I Hsu, Deng-Chyang Wu, Keng-Liang Wu, Chao-Cheng Huang, Ji-Chen Ho, Johannes Ring, Wen-Chieh Chen

**Affiliations:** ^1^Division of Hepato-Gastroenterology, Department of Internal Medicine, Kaohsiung Chang Gung Memorial Hospital, Chang Gung University College of Medicine, 123 Ta-Pei Road, Niao-Sung District, Kaohsiung 833, Taiwan; ^2^Division of Gastroenterology, Department of Internal Medicine, Kaohsiung Veteran General Hospital and National Yang-Ming University, Kaohsiung 813, Taiwan; ^3^Cancer Center, Division of Gastroenterology, Department of Internal Medicine, Kaohsiung Medical University Hospital, Kaohsiung Medical University, Kaohsiung 807, Taiwan; ^4^Department of Pathology, Kaohsiung Chang Gung Memorial Hospital, Chang Gung University College of Medicine, Kaohsiung 833, Taiwan; ^5^Department of Dermatology, Kaohsiung Chang Gung Memorial Hospital, Chang Gung University College of Medicine, Kaohsiung 833, Taiwan; ^6^Department of Dermatology and Allergy, Christine Kuehne-Center for Allergy Research and Education (CK-CARE), Technical University of Munich, Biedersteiner Str. 29, 80802 Munich, Germany

## Abstract

*Background and Study Aims*. The association between *Helicobacter pylori* (*H. pylori*) and chronic spontaneous urticaria (CSU) remains controversial. This study explored the role of *H. pylori* in CSU among different virulent genotypes patients. *Patients and Methods*. Patients infected by *H. pylori* were sorted into two groups as group A (with CSU) and group B (without CSU). The tissue materials were taken via endoscopy for polymerase chain reaction study to determine virulence factors. After *H. pylori* eradication therapy, the eradication rate and response of urticaria were evaluated by using C^13^-UBT and a three-point scale (complete remission, partial remission, or no improvement). *Results*. The results were comparable between patients of groups A and B in terms of *H. pylori* infection rates and eradication rate. Longitudinal follow-up of 23.5 months showed complete remission of urticaria in 63.6% but no improvement in 36.4% of the patients after *H. pylori* eradication. *H. pylori* infected patients with different virulence factors such as cytotoxin-associated gene A, vacuolating cytotoxin gene A signal region and middle region have similar remission rates for CSU. *Conclusions*. Current study suggests that *H. pylori* may play a role in the development and disease course of CSU but may be irrelevant to different virulent genotypes.

## 1. Introduction

Chronic spontaneous urticaria (CSU), defined as spontaneous occurrence of wheal and/or angioedema lasting for a period of longer than 6 weeks, is a common and often frustrating problem, affecting up to 1 percent of the general population [1, 2]. The causes of CSU are numerous; however, in at least 80–90% of the patients, the etiology is undetermined [2, 3]. Recent data show that about 30% of the affected patients may have functional autoantibodies [4]. On the other hand, *Helicobacter pylori* (*H. pylori*) infection is probably the most common chronic bacterial infection in humans, with the prevalence rate in general population estimated to be around 50% in developing countries [5]. It has been generally accepted that *H. pylori* infection plays an etiologic role in the development of chronic active gastritis, peptic ulcer disease, gastric malignancy, and low-grade gastric mucosa-associated lymphoid tissue lymphoma [5–8]. *H. pylori* is genetically highly diverse, and several genotypes have been identified to associate with severe gastric mucosal inflammation [9]. Cytotoxin-associated gene A (cagA) and vacuolating cytotoxin gene A (vacA), the two most important virulence factors of *H. pylori* [9], have been reported to enhance its pathogenicity [10] while cagA is related to peptic ulcer and gastric malignancy in certain populations [11, 12]; vacA can induce host cell vacuolation and eventually cell death [11]. A high degree of sequence variability exists in the vacA gene, with its signal and middle region being classified into s1/s2 and m1/m2 subtypes, respectively [13]. The s1 subtype and m1 subtype have been linked to more severe gastrointestinal manifestations [14].

A potential association between CSU and *H. pylori* infection of the upper gastrointestinal tract has been proposed, but the studies so far showed controversial results [15–18]. Moreover, little is known about the association between the genotypes of *H. pylori* and CSU [15]. This study aimed to explore the potential role of *H. pylori* in the development and disease course of CSU among the different virulent genotypes of patients.

## 2. Patients and Methods

### 2.1. Study Design

From August 2008 to July 2009, 25 patients (age 27–68 years, mean = 45.5 years, female/male = 13/12) diagnosed as CSU with unremarkable findings in allergy diagnostics (basis examination) were recruited from the Dermatology Outpatient Department of Chang Gung Memorial Hospital-Kaohsiung, Taiwan [1]. The duration of CSU ranged between 6 and 360 months with a median of 12 months. Only six of them (6/25, 24%) suffered from upper gastrointestinal symptom. All of them received a C^13^-urea breath test (C^13^-UBT). Infection of *H. pylori* was diagnosed by a positive C^13^-UBT test and sorted as group A (*n* = 14). Meanwhile, 24 patients (age 18–83 years, mean = 41.5 years, female/male = 13/11) with gastrointestinal symptoms but without urticaria/pruritus were enrolled from the Gastroenterology Department for C^13^-UBT examination, and the infected patients were categorized into group B. All the C^13^-UBT-positive patients underwent upper gastrointestinal endoscopy, using a GIF XQ 240 endoscope (Olympus Optical Company, Tokyo, Japan), and tissue biopsies were taken from the gastric antrum and body (*n* = 14). Criteria for exclusion included (a) ingestion of antibiotics, bismuth, or proton-pump inhibitors within the prior 4 weeks, (b) use of nonsteroidal anti-inflammatory drugs within the prior 4 weeks, (c) patients with previous gastric surgery, (d) the coexistence of serious concomitant illness (e.g., decompensated liver cirrhosis or uremia), (e) pregnant women, and (f) those who refused endoscopic examination and subsequent *H. pylori* eradication. *H. pylori *infection was defined as positive results by a positive C^13^-UBT test.

All the biopsied specimens were stored in 70% ethanol in Eppendorf tubes at −80°C until processed for polymerase chain reaction (PCR) examination, in which the tissue specimens were homogenized with a sterile micropestle, and the DNA was extracted and purified using a commercial kit following the tissue protocol of the manufacturer (QIAGEN QIAamp DNA mini kit, Hilden, Germany). To detect the virulence factors of *H. pylori*, PCR studies with three respective species-specific primer sets were designed to amplify highly conserved regions within the genes encoding cagA and vacA (s and m regions) [19].

### 2.2. Treatment Allocation

All the infected patients then received oral eradication therapy comprising esomeprazole (40 mg twice daily), clarithromycin (500 mg twice daily), and either amoxicillin (1 gm twice daily) or metronidazole (500 mg twice daily) for patients with penicillin allergy in the history [12]. Esomeprazole and amoxicillin were taken one hour before breakfast and dinner, clarithromycin and metronidazole twice daily after breakfast and dinner. To assess eradication efficacy, a repeated C^13^-UBT was performed to each patient at six weeks after the end of anti-*H. pylori* therapy. The effectiveness of eradication therapy on CSU was assessed three months later after treatment, using a three-point rating scale, that is, complete remission, partial remission (50% or more), or no improvement. The differences in cagA, vacA s, and vacA m of *H. pylori* between patients of group A and group B as well as the differences in clinical course of CSU before and after eradication therapy relating to the various virulent factors were analyzed. The study was approved by the ethic committee of Chang Gung Memorial Hospital-Kaohsiung, Taiwan (no. 95-1314B), and signed informed consent was obtained from all the participants.

### 2.3. Statistical Analysis

Continuous variables, given as means and standard deviations (SD), were analyzed using the Mann-Whitney *U* test. Categorical variables, given in total and as percentages, were analyzed using the Chi-square test or Fisher exact test. Two-sided *P* values of <0.05 were considered significant. All the statistics were performed using SPSS (WIN version 15.0).

## 3. Results

**Figure 1:**
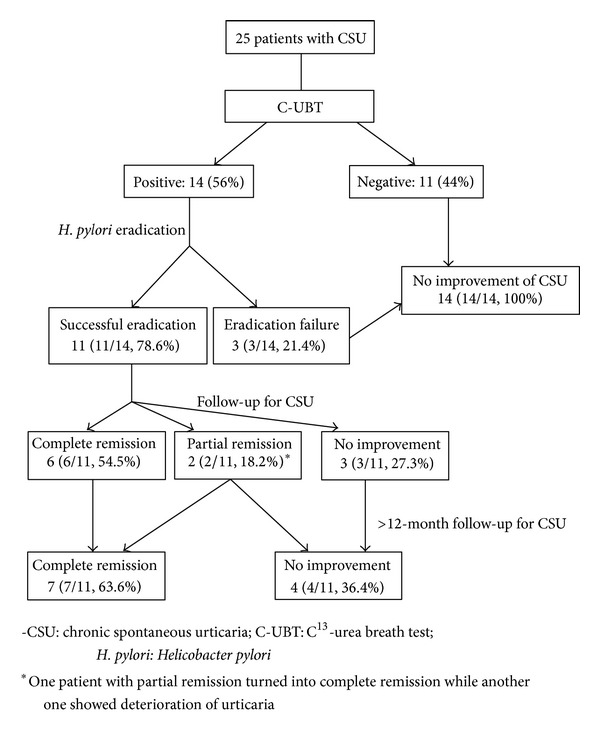
Allocation diagrams of patients with chronic spontaneous urticarial.

Allocation diagrams of patients with chronic spontaneous urticaria are summarized in Figure 1. Demographic data of *H. pylori*-infected patients with or without chronic spontaneous urticaria (CSU) was summarized in Table 1. In group A, clinical follow-up of 11 successfully treated patients three months later revealed complete remission of urticaria in 54.5% (6/11), partial remission in 18.2% (2/11), and no improvement in 27.3% (3/11). In longitudinal follow-up studies for a duration of 12–29 months after *H. pylori *eradication (median = 23.5 months), complete remission was found in 63.6% (7/11) and no improvement in 36.4% (4/11) of the patients. One patient with partial remission turned into complete remission while another one showed deterioration of urticaria. All the three patients in group A who failed *H. pylori *eradication showed clinically persisting urticarial symptoms. The duration of urticaria was 14.9 ± 5.8 months in the treatment responders as compared to 15.1 ± 8.8 months in the nonresponders. However, there was no statistical difference observed in the clinical response rates of CSU between the treatment success and treatment failure patients (7/11, 63.6% versus 0/3, 0%, *P* = 0.193, Fisher's exact test). None of the patients in group B developed urticarial lesions in an average follow-up of 14.1 months.

**Figure 2:**
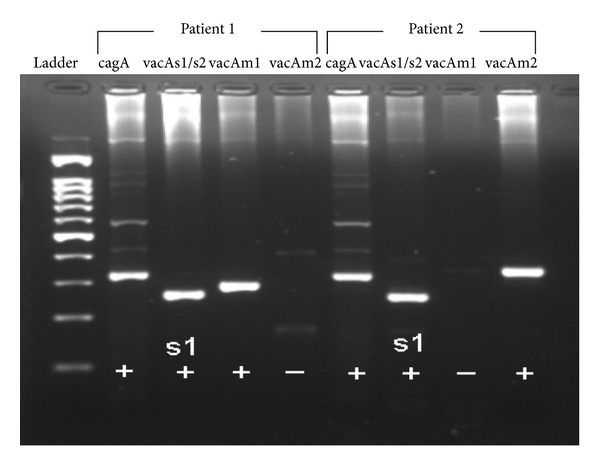
Results of PCR study showed the amplification products of *Helicobacter-*specific virulence factors cagA (324 bp), vacA s1 (259 bp), vacA s2 (286 bp), vacA m1 (290 bp), and vacA m2 (352 bp) such as patient 1 with CSU and positive genotypes cagA and vacA s1m1 and patient 2 (control group) with positive expression of cagA and vacA s1m2 but without CSU.

Determination of virulence factors via PCR study showed the size of the amplified products as follows: cagA (324 bp), vacA s1 (259 bp), vacA s2 (286 bp), vacA m1 (290 bp), and vacA m2 (352 bp) (Figure 2). The cagA genotype was detected in 11/14 (78.6%) in group A and 13/14 (92.9%) in group B (*P* = 0.596). The ratio of s1 to s2 alleles in vacA was 11 (78.6%) to 3 (21.4%) in group A and 13 (92.9%) to 1 (7.1%) in group B (78.6% versus 92.9%, *P* = 0.596). The ratio of m1 to m2 alleles was 6 (42.9%) to 8 (51.7%) in group A and 2 (14.3%) to 12 (85.7%) in group B (42.9% versus 14.3%, *P* = 0.209). The expression of cagA, vacA (s1/s2), and vacA (m1/m2) did not differ between patients in group A and group B (*P* = 0.596, 0.596, and 0.209, resp.) (Table 2). In analysis of the association between virulence genotypes and therapeutic response of urticaria (group A), the cagA genotype was detected in 5 (71.4%) of 7 CSU patients with remissions (complete and partial) and 6 (85.7%) of 7 patients without improvement (*P* = 1.0). The s1/s2 ratio of vacA genotype was 5 (71.4%) to 2 (28.6%) in remission patients and 6 (85.7%) to 1 (14.3%) in nonremission patients (*P* = 1.0). The ratio of m1 to m2 alleles in vacA was 3 (42.9%) to 4 (57.1%) with remission of CSU posttreatment and 3 (42.9%) to 4 (57.1%) with persisting CSU (*P* = 1.0). As summarized in Table 2, there was no significant difference observed between CSU patients in remission and nonremission status after successful *H. pylori *eradication treatment for the expression of virulence factors such as cagA, vacA (s1/s2), vacA (m1/m2), vacA s1m1, or (cagA + vacA) s1m1.

## 4. Discussion

A potential association between CSU and *H. pylori* infection of the upper gastrointestinal tract has been proposed, but the studies so far showed controversial results not to mention the relevance to the genotypes of *H. pylori *[15–18]. In current study, the prevalence rate of *H. pylori* infection of 56% in our CSU patients which is rational as reported in other studies varied from 47% to 80% [20–23]. All of these studies, including ours, also observed that the prevalence of *H. pylori* infection between patients with and without CSU is similar. Moreover, follow-up of the 14 infected patients with gastric complaints but without skin problems (group B) for an average of 14.1 months did not find a later development of CSU. However, clinical regression of CSU was observed after a successful eradication of *H. pylori*, among 17.6% to 88% of these patients [20–23]. 58.3% of our CSU patients had a long-term complete remission of their CSU after an effective eradication therapy. Given that a relatively low reported spontaneous remission rate of 6% in the natural course of CSU persisting for more than 6 months [24], current study demonstrated an apparent benefit from *H. pylori* eradication therapy for CSU patients and suggested that a possible etiopathogenetic role of *H. pylori *may exist. 

*H. pylori* species is genetically highly diverse [9]. Differences in the genotypes of bacterial virulence factors can induce variable degrees of gastric inflammation and elicit different clinical manifestations in gastrointestinal tract. The strains have been categorized into type I with expression and type II without expression of the virulence factors [25]. Type I *H. pylori* can cause mucosal damage by stimulating gastric epithelial cytokine responses and produce a variety of other factors that determine the local inflammatory response [26]. Among the well-characterized virulence factors of type I strains, cagA, vacA-s, and vacA-m have been reported to enhance pathogenicity of *H. pylori *[10]. CagA-positive strains induce a higher production of proinflammatory cytokines in the gastric mucosa and are linked with an increased risk of peptic ulcer disease, gastric atrophy, and gastric cancer in certain populations [27, 28]. VacA, which is produced by 50–60% of *H. pylori* strains, causes fusion of the endocellular lysosomes leading to a consequent reswelling of the gastric epithelium cells [27]. *H. pylori *bacteria carrying vacA*-*s1 and vacA-m1 subtypes have also been related to more severe clinical manifestations [9, 27].

There is proposal of potential association between CSU and *H. pylori* infection of the upper gastrointestinal tract [15–18], but only one study reported the clinical relevance between virulence factors of *H. pylori* and CSU [15]. Fukuda and his colleagues found a high incidence of cagA expression in CSU patients (100%, 13/13) and similarly in control group (100%, 26/26) [15]. This was the same to current study. Longitudinal follow-up (range 12–29 months, median 23.5 months) showed complete remission of urticaria in 63.6% (7/11) and no improvement in 36.4% (4/11) of our patients after *H. pylori* eradication. In addition, we had shown that the expression of other virulence factors did not differ between patients with and without CSU. *H. pylori* infected patients with different virulence factors such as cytotoxin-associated gene A, vacuolating cytotoxin gene A signal region and middle region had similar remission rates for CSU. Moreover, among CSU patients, the genotypes of *H. pylori* virulence factors did not correlate with the onset age, gender difference, or the response to bacterial eradication therapy. 

*H. pylori *colonizes the gastric mucosa in approximately half of the world's population, but only a minority (10–20%) of the infected individuals develop clinical manifestations, most commonly with gastrointestinal disorders [7, 27]. There were debates on the clinical association of *H. pylori* with certain dermatological disorders which includes issues such as CSU, rosacea, psoriasis, or immune thrombocytopenic purpura [25, 29]. Like peptic ulcer disease, rosacea may be triggered by the more virulent type I strain of *H. pylori* [30]. It is also believed that many environmental, bacterial, and host-related factors can influence the course of infection. The cagA protein of the more virulent strain stimulates the gastric epithelium to secret greater amounts of inflammatory cytokines such as IL-8, IL-1, TNF-*α*, interferon-*γ*, leukotrienes, and platelet-activating factors [30]. On the other hand, it is suggested that *H. pylori* infection may facilitate penetration of allergens with induction of an IgE response to certain common alimentary antigens thereby enhancing the development of food allergy [31]. Infection with *H. pylori* has been shown to increase antigen absorption across the cultured digestive epithelium *in vitro* and across the gastric mucosa of mice* in vivo* [32, 33]. Nevertheless, it is still unclear whether the immunologic reactions induced by *H. pylori* can directly elicitate the mast cell degranulation leading to urticarial formation.

Current study encounters some limitations. First, the sample size is relatively small so bias may still exist. Second, there is no matched control group for this study which should be untreated from *H. pylori* infections. The bottom line is that it is practically unethical not to treat a diagnosed infection. Third, there are other virulence factors of *H. pylori* which were not studied, such as iceA, babA, flaA/flaB (the genes for flagellins), and ureA/ureB (urease-encoding genes). As we know, iceA is induced by contact with epithelium; babA is associated with binding to blood-group antigens [34, 35]. However, unlike cagA, vacA-s1, and -m1, iceA1 and babA2 which are associated with a more severe gastrointestinal manifestation [35, 36], flaA/flab and ureA/ureB genotypes are of less clinical significance [37].

## 5. Conclusions

Current study suggests that *H. pylori* may play a role in the development and disease course of CSU. Different virulent genotypes of *H. pylori* may be irrelevant to the remission of CSU after eradication. However, it remains to be determined whether a quantitative effect of the examined virulence factors may exist or other virulent and nonvirulent genes of *H. pylori* may play a role in the pathogenesis of CSU. A better understanding of the bacterial virulence factors and the corresponding host immune response is still needed to further clarify the pathogenic role of *H. pylori* in certain groups of patients with CSU. 

## Figures and Tables

**Table 1:** Demographic data of *H. pylori*-infected patients with or without chronic spontaneous urticaria (CSU).

	Group A (with urticaria) *N* = 14	Group B (without urticaria) *N* = 14	*P* value
Age (years)	41.2 ± 11.7	47.8 ± 7.6	0.114
Gender (male/female, %)	50	35.7	0.704
Success rate of *H. pylori* eradication (%)	11 (78.6)	10 (71.4)	1.0
Genotype			
cagA (%)	11 (78.6)	13 (92.9)	0.596
vacAs1/s2 (%)	11 (78.6)/3 (21.4)	13 (92.9)/1 (7.1)	0.596
vacAm1/m2 (%)	6 (42.9)/8 (51.7)	2 (14.3)/12 (85.7)	0.209

**Table 2:** The expression of virulence factors for CSU patients in remission and nonremission status after successful *H. pylori* eradication treatment.

	Remission (*N* = 7)	Nonremission (*N* = 7)	*P* value
Genotype			
CagA	5 (71.4)	6 (85.7)	1.0
VacAs1/s2	5 (71.4)/2 (28.6)	6 (85.7)/1 (14.3)	1.0
VacAm1/m2	3 (42.9)/4 (57.1)	3 (42.9)/4 (57.1)	1.0
VacA s1m1	2 (28.6)	3 (42.9)	0.5
CagA + VacA s1m1	2 (28.6)	3 (42.9)	0.5
